# Case of a 35-year-old female with cellulitis on lower extremities with severe preeclampsia in pregnancy: a rare clinical image

**DOI:** 10.11604/pamj.2024.49.61.45391

**Published:** 2024-10-30

**Authors:** Switi Jawade, Archana Teltumde

**Affiliations:** 1 Department of Obstetrics and Gynaecology Nursing, Smt Radhikabai Meghe Memorial College of Nursing, Datta Meghe Institute of Higher Education and Research (DU) Sawangi Meghe, Wardha, India

**Keywords:** Cellulitis, pregnancy, skin infection, *Staphylococcus* bacteria, preeclampsia

## Image in medicine

Cellulitis is caused by a bacterial infection of the skin. Most often, the bacteria responsible for the infection is *Staphylococcus*. Cellulitis starts on broken, swollen, or cracked skin. Typically, the infections are found on feet and legs, but they can start anywhere on the body. When pregnant, swelling associated with fluid retention can increase the risk of developing cellulitis. We here report the case of a 35-year-old gravida 2, abortion 1 at 36+3 weeks gestational age with severe pre-eclampsia with cellulitis female patient, who came to the hospital with a history of amenorrhea for 9 months, pain in her abdomen for one day, leaking per vaginal since morning with the complaints of swelling over labia, tinea lesion present on bilateral upper limb and neck region, fluid-filled blister over the left lower extremities, pitting edema present up to mid-thigh. The patient had taken treatment from a private hospital for the sixth month of pregnancy in view of raised blood pressure (BP) to 190/80 mmHg on a tab. Labet 100 mg once a day (OD). On physical examination pulse 110/min, resp-16/min, blood pressure 180/60 mmHg. On general examination her condition was poor. On abdominal examination per abdomen wall edema was present, fundal height could not be assessed, fetal heart sound (FHS) dull sound, severe fetal bradycardia/100 beats per minute (Bpm). Gynecological examination on per vaginal vulva edema presented, cervix 4 cm dilated, 25% effaced, station (-2) presenting part vertex (PPVX), membrane rupture. On blood test hemoglobin 10gm%, mean corpuscular hemoglobin concentration (MCHC) 32.9, mean corpuscular volume 109.9, total red blood cell count 2.78, total white blood cell count 7500, total platelet 1.04, random blood sugar glucose-plasma random 42. The patient was taken for emergency cesarean section, a stillborn male baby was delivered with 2.1 kg.

**Figure 1:**
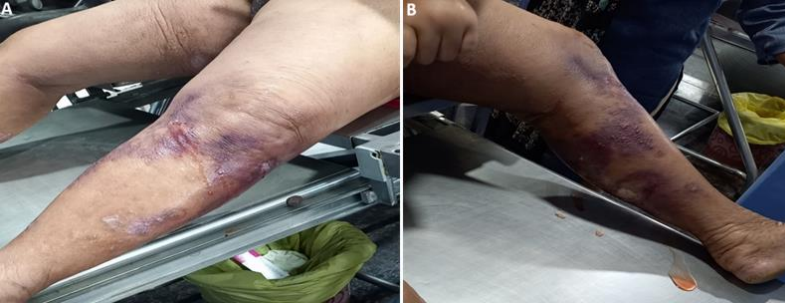
A) fluid filled blister over the lower extremities; B) bacterial skin infection with discolouration skin of lower extremities

